# Assessment of knowledge, attitude and practice regarding intellectual property rights among dental task force attending private dental colleges in Navi Mumbai: a cross-sectional study

**DOI:** 10.25122/jml-2020-0103

**Published:** 2021

**Authors:** Vaibhav Kumar, Venetia Aranha, Nikhil Bhanushali, Romi Jain, Swarali Atre, Shishir Singh

**Affiliations:** 1. Department of Public Health Dentistry, TPCT'S Terna Dental College, Navi Mumbai, India; 2. Department of Conservative Dentistry and Endodontics, TPCT’S Terna Dental College, Navi Mumbai, India

**Keywords:** intellectual property, license, patent, trademarks, copyright, IP – Intellectual Property, IPR – Intellectual Property Right, IPRs – Intellectual Property Rights

## Abstract

Intellectual property rights such as Copyright, Trademark, Patents and Trade secrets etc. help us to gain some protection against certain inventions by acknowledging the founder. In today's industry it is agreed that Intellectual Property Rights has a big role to play. This current study envisages the knowledge, attitude, practice regarding Intellectual Property Rights among dental task force attending private dental colleges.The survey was conducted among students of which were Interns, Post Graduates, faculty members and other dental surgeons attending private dental colleges in Navi Mumbai. The subjects of this study comprised of a total of 1020 students, faculty members and other dental surgeons from five different private dental institutes. The survey includes closed ended questions. Data analysis was performed using SPSS software version 17. Explaining calculations were used to summarize all the answers. A total of 889 students, faculty and other dental surgeons from private dental colleges responded. Results showed that about 83.5% believe the statement “Articles and other publications are protected by copyright.” 66.6% of participants would select trademark in order to protect their clinic or organization name. About 38.7% were aware of the term Intellectual Property Rights. It also suggests that only 10.9% have attended any seminar/conference pertaining to IPR. The students and faculty members have an overall sense of eagerness to learn and gain more knowledge based on IPR. Thus conducting more workshops and seminars based on IPR should be encouraged.

## Introduction

Globalization and advancement in digital technologies have bought about changes in the rapidly emerging and evolving cognitive field as well as the hands-on department. This has led to the increase in the importance of intellectual property rights (IPRs), which encourages fair use and preempts plagiarism. IPRs are artistic, creative ideas resulting from the human brain applying art and design and inventing something unique based on the community’s pursuit to add property [1]. These rights are assigned to creators for a specific period when the builder acquires them in their work [2]. They include copyright, trademark, design, trade secrets and patents, which provide benefits to creative efforts by acknowledging them [3].

In the era of recession, many international companies dominated the Indian pharmaceutical market. Drugs were exported at a high cost making India one of the world’s highest-priced nations. It was observed that the old Indian Patents and Designs Act, 1911 did not serve the needs of the Indian people [5]. As the Indian patent system was a process run by The “Process patent”, the process of transitioning to the “patent system” was expected to be a nightmare for the pharmaceutical industry, and the initial reaction was overwhelming [6]. An IPR license is often contractual and crucial to avoid unfair practice wherein the licensee is authorized by the licensor to perform tasks that otherwise would be illegal [7].

Patents protect the dental or medical diagnostic products from being used commercially without the inventor’s consent. Class-grade patents form the basis of the country’s scientific, industrial, and economic growth [6]. A technological strategy employed is “evergreening,” which uses “one-second patent,” which are small formulations or other modifications of a patent that tend to extend the lifetime of a patent. The Indian Patent Act contravenes the evergreening measures by introducing section 3 (d), which distinguishes between “new discoveries” and explicitly defines a patent. Initially, ‘industrial property’ only protected certain rights of which encompass patents, trademarks, and industrial design. The term ‘property ownership’ protects more than one right, thus expanding its meaning and promoting technological advancement in several ways [8]. An intellectual property (IP) developer may own, control, and be rewarded for its use thus, promoting and benefiting all [9]. IPRs are licensed like any other property [10], and IP infringing laws avert the breaching of unembodied ideas. Plagiarism will not decline unless strict infringing laws are applied. This is problematic, and one should shed some light to help researchers and publishers to take advantage of it to avoid copyright infringement [11]. 

Copyright offers protection to written text, drawings, or any physical creation by storing it in the computers [12]. Under copyright law, one of the most important restrictions is the doctrine of “fair use.” This doctrine is limited and distinct from the exclusive right granted to the author of the creative work. It allows restricted use of copyrighted material without the consent of the copyright holders [13]. Many countries have established national regimes to provide protection from IPR under its law. Except in the case of copyrights, protection provided to the manufacturer in a country (such as India) or a region (such as the European Union) is limited to the area where the protection is sought and is not applicable in other countries or regions. For example, a patent acknowledged in India is not valid in the United States of America [14].

Furthermore, IPR laws are at different implementation stages in India, but there is no distinct rule for protecting anonymity for trade secrets or confidential information [15]. IP should be a collective action of building, managing, and selling the idea and is considered a financial resource. When the transaction of an idea begins, special IP will become the foundation [16]. It is a right granted by the government empowering others to exclude, use or practice the innovative idea/method [17]. To avoid corrupt practices in the dental industry, such as fraudulent imitation of an organization name, surgical procedures IPR is the need of the hour. Dental students and doctors should be aware of the benefits they have.

Strikingly revealing, thorough scientific literature search yielded results that there was not a single study published on the awareness and utilization of IPR or such constructs among the dental task force. Hence, the current study aims to assess the knowledge, attitude, and practices of dental professionals about IPR.

## Material and Methods

This study employed an observational, cross-sectional study design and was carried out in accordance with the Strengthening the Reporting of Observational Studies in Epidemiology (STROBE) guidelines to collect prevalent data about the knowledge, attitude and practices pertaining to intellectual property rights among the dental task force of five private dental colleges in Navi Mumbai, India [18]. The duration of this study was 3 months, from January 2020 to March 2020. 

The participating dental task force comprised interns, postgraduates and faculty members of five private dental colleges viz Institute A, B, C, D and E located in Navi Mumbai. Those present on the day of the study and willing to give informed consent were included and those absent and unwilling to give their consent were excluded from the study.

A pilot study was conducted among 50 participants to check for the flaws and feasibility of the study. A structured English language questionnaire that subsumed 24 close-ended questions containing 14 general questions based on IPR, 3 questions based on copyright, 5 questions based on trademark and 2 questions based on patents was curated similar to that used in a prior study about IPR awareness [2]. Prior to the finalization of this survey tool, its content validity was assessed by a panel of six subject experts who expressed their opinions in order to calculate the mean Lawshe’s Content Validity Ratio (CVR), which was discerned to stand at 0.92. On assessing the face validity, 92% found the survey tool to be easy and comprehendible. This ascertained that the designed questionnaire assessed the desired qualities that it intended to encapsulate and measure within its ambit. Internal consistency estimates of reliability using Cronbach’s Alpha were computed on domain-specific items to confirm the development of subscales of knowledge, attitude and practice of the questionnaire, and the Cronbach’s coefficient of 0.91 showed high internal reliability.

**Figure 1.**
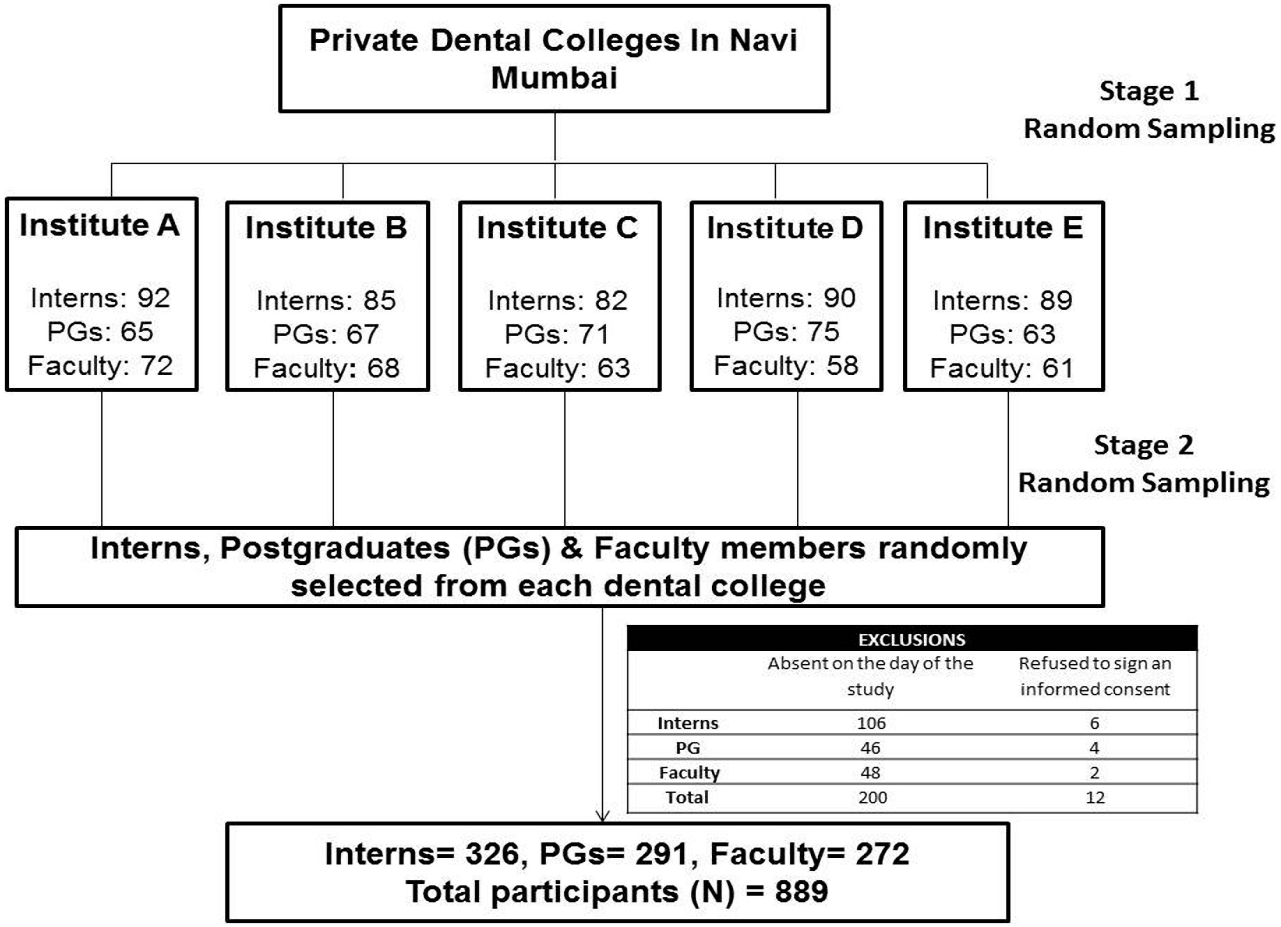
Study flowchart.

On the basis of the pilot study, using the G* Power Statistical Software (version 3.1.9.2), a sample size of 889 was calculated. A multi-stage random sampling technique was incorporated for sample acquisition, which has been illustrated in Figure 1.

The data obtained were tabulated and analyzed statistically using the SPSS software, version 17. The normality of the data was assessed prior to analysis using the Shapiro-Wilk’s test/Kolmogorov-Smirnov test. Descriptive analysis through frequency distribution was calculated, and the Chi-Square test was applied. A probability of less than 0.05 was considered significant.

## Results

**Figure 2.**
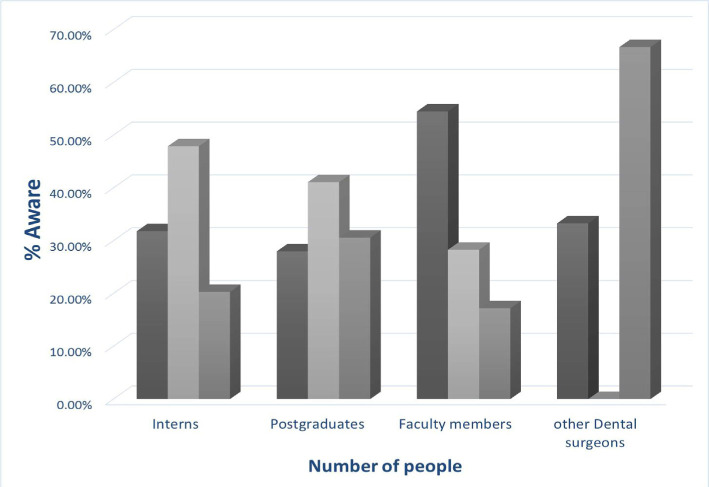
Awareness regarding intellectual property rights.

The study demonstrated the explicit facts regarding the awareness of IPR; the interns accounted for 31.8%, postgraduates – 28.3%, faculty members – 54.5%, other dental surgeons – 33.3% who were unaware of the term and a significant difference (p<=0.001) was noted (Figure 2). This was the major finding.

Furthermore, there was a similar response between the interns (41.4%), postgraduates (41.1%), and faculty members (42.8%) who opted for copyright in order to protect innovation/working model/device (Table 1). Consequently, a significant difference (p≤0.001) was seen among interns (63.0%) and postgraduates (75.0%), disclosing the fact that they would select a trademark for their organization name (Table 1), indicating their knowledge regarding trademark.

**Table 1:** Knowledge and practice regarding intellectual property rights.

Question / Statement	**Response**	**Individual responses of participants in percentages (%)**				**P-value**
		**Interns**	**Postgraduates**	**Faculty members**	**Other Graduates and dental surgeons**	
Are you aware of any laws for protecting the laws of consumers?	Yes	55.3%	48.3%	68.4%	100%	<0.001*
Have you been a victim of deficiency in service/unfair trade practices?	Yes	21.3%	33.9%	32.3%	0.0%	<0.001*
Are you aware of copyright on articles and publications?	Yes	82.1%	85.0%	85.9%	33.3%	<0.001*
Are you aware of the public search registered portals for trademarks/patents?	Yes	35.7%	35.0%	39.7%	33.3%	<0.001*
Are you aware that telling people about an innovation before applying for a patent could lead to an unsuccessful application?	Yes	92.1%	91.8%	93.9%	66.7%	<0.001*
Are you aware that once a trademark is registered no one else will be able to register the same trademark?	Yes	93.8%	96.1%	100%	66.7%	<0.001*
Are you aware that the symbol ® means that a name or logo is protected?	Yes	95.8%	96.7%	94.9%	66.7%	<0.001*
Would you choose trademark to establish IPR over your clinic/organization name?	Yes	63.0%	75.0%	67.3%	33.3%	<0.001*
Would you choose copyright to establish IPR over information/education/ communication material that you have curated?	Yes	48.1%	53.9%	48.8%	66.7%	<0.001*
Would you choose copyright over an innovation/working model/device?	Yes	41.4%	41.1%	42.8%	0.0%	0.003*

* Chi square test applied, <0.05 indicates statistical significance; IPR: intellectual property right.

However, the majority of the respondents showed the desired need for seminars and workshops regarding IPR, since interns (87.3%), postgraduates (83.9%), faculty members (86.5%), and other dental surgeons (66.7%) responded positively (Table 2).

**Table 2:** Attitude regarding intellectual property rights.

Question/Statement	**Response**	**Individual responses of participants in percentages (%)**				**P-value**
		**Interns**	**Postgraduates**	**Faculty members**	**Other Graduates and dental surgeons**	
Do you think the nature of IPR is a right?	Yes	54.8%	73.3%	71.4%	0.0%	<0.001*
Do you think the base of its use is that it can be sold, purchased or registered?	Yes	79.4%	71.7%	73.1%	66.7%	<0.001*
Do you think the protection of IPR is through law and registrations?	Yes	73.9%	77.8%	78.5%	0.0%	<0.001*
Do you think the description of IPR is that it is only for business and monopoly?	Yes	37.7%	45.6%	41.4%	53.3%	<0.001*
Do you think the protection of IPR is to stop exploitation?	Yes	28.0%	32.8%	20.5%	0.0%	<0.001*
Do you think the use of IPR is for study and research, to stop plagiarism and scientific innovation?	Yes	75.9%	77.8%	95.6%	33.3%	<0.001*
Do you think trademark is important to intent the quality of goods and to mark the goods and services?	Yes	74.4%	80.6%	81.1%	0.0%	<0.001*
Do you think your organization name should be protected by a trademark?	Yes	46.4%	59.4%	65.3%	66.7%	<0.001*
Has your company ever sort advice on IPR?	Yes	33.0%	22.2%	25.6%	0.0%	0.033*
Do you seek an impending need for IPR sensitization at your institute or faculty?	Yes	28.0%	28.9%	36.4%	66.7%	<0.001*
Have you attended any seminar/conference pertaining to IPR?	Yes	14.1%	7.2%	9.1%	0.0%	0.089*
Do you wish to attend any seminar / conferences pertaining to IPR in the future?	Yes	87.3%	83.9%	86.5%	86.2%	0.003*

*Chi square test applied, <0.05 indicates statistical significance; IPR: intellectual property right.

Although interns (75.9%), postgraduates (77.8%), and other dental surgeons (33.3%) lacked knowledge on whether the use of IPR is to stop plagiarism, the faculty members (95.6%) were cognizant of this (Table 2), indicating the need for imparting education in the field of dentistry regarding IPR.

## Discussion

Awareness regarding the knowledge of different types of IPR in the health care sector is crucial. IPR is a sine qua non to identify, sell, and defend an innovation [4]. This current study illustrates the knowledge, attitude and practice about the awareness of IPR among interns, postgraduates, faculty members and dental surgeons in five different institutes in Navi Mumbai, accounting for a total of 889 participants. A similar study was conducted regarding the awareness of IPR among the postgraduate (PG) and Ph.D. law students in Lucknow, India, and contains analogous questions. However, the study conducted showed that law students were somewhat aware regarding copyright, they lacked a great deal of knowledge regarding patents, and had some misconceptions regarding trademark [19]. 

The fact that the cognizance among the interns – 31.8%, postgraduates – 28.3%, and faculty members – 54.5%, other dental surgeons – 33.3% (Figure 1) was little in accordance with IPR and the need to attend seminars and workshops among interns (87.3%), postgraduates (83.9%), faculty (86.5%) and other dental surgeons (86.5%) was large, is the major find of the study.

In IPR law, copyright safeguards the definitions of ideas, including information, planning and dissemination of patient/medical information and medical books. Our study revealed that interns (82.1%), postgraduates (85.0%), faculty members (85.9%), and other dental surgeons (33.3%) showed a positive response with respect to the copyright protection on articles, which was statistically significant (p≤0.001) as to the survey conducted by Ahmed *et al.* on law students (20%) [19]. Interns (48.1%), postgraduates (53.9%), faculty members (48.8%), and dental surgeons (66.7%) would choose copyright in order to secure the information they have curated. Also, about 41.4% of interns, 41.1% of postgraduates and 42.8% of faculty members would choose copyright in order to safeguard the innovation/working model they have invented. This poor response might be ascribed to the fact that the dental industry is not well versed with copyright laws.

Trademark describes features that distinguish services. It usually provokes healthy competition, helps consumers with information about options, making these unique signs available visually [13]. Trademarks are brand names for healthcare-related services that protect the designs of various articles and products used in the healthcare industry.

A stark contrast was seen regarding the importance of trademark between our study and the study conducted by Ahmed *et al.* among law students who reported awareness in 44% of cases. In contrast, in this study, interns, postgraduates and faculty members showed awareness in 74.4%, 80.6%, and 81.1% of cases, respectively [19]. Furthermore, it was seen that interns (93.8%), postgraduates (96.1%), faculty members (100%), and other dental surgeons (66.7%) showed a relatively higher response about the trademark registration. The interns (95.8%), postgraduates (96.7%), faculty (94.9%), and other dental surgeons (66.7%) showed a positive attitude to the fact that the symbol of ® is used to protect the name/logo. The three categories of participants showed a low response (interns – 63.0%, faculty members – 67.3%, dental surgeons – 33.3%) as compared to the postgraduates – 75.0% about the use of a trademark to protect their organization name and a statistically significant difference was seen. This difference might be attributed to the fact that interns and faculty members have lesser knowledge than postgraduates on trademark services and registration.

Patents protect the products from being used commercially. An overwhelming response was obtained by the three groups of respondents (interns – 92.1%, postgraduates – 91.8%, faculty – 93.9%) as compared to dental graduates (66.7%), which were statistically significant (p≤0.001) regarding patents. In contrast, the study conducted by Ahmed *et al.* showed that only half of the law students (50%) shared the same opinion. [19] On evaluation, the nature of IPR was considered as a right by interns (54.8%), postgraduates (73.3%), and faculty members (71.4%). A similar response (50%) was obtained by law students (50%) [19].

In addition, 35.7% of interns, 35.0% of postgraduates, 39.7% of faculty members and 33.3% of dental surgeons were informed concerning the public search/registered portals. This indicates that only one-third of the dental professionals were aware of the registered portals and should be encouraged to attend seminars regarding this topic. However, our study showed that interns (37.7%), postgraduates (45.5%), faculty members (41.4%), and other dental surgeons (53.3%) agree to the fact that the description of IPR is only to create business and monopoly. In contrast, only a few of the law students (6%) that participated in the study conducted by Ahmed *et al.* agreed to the statement [19]. This significant discrepancy might be due to dental professionals not being educated regarding the aspects of IPR. 

Consequently, the study demonstrated that dental surgeons (33.3%) had lesser knowledge than the remaining three respondent groups (interns – 75.9%, postgraduates – 77.8%, faculty members – 95.6%) with a statistically significant difference (p≤0.001) in regard to the usage of IPR and only a few law students from the study conducted by Ahmed *et al.* responded positively to this [19]. The participants of our study showed a strikingly high response (interns – 73.9%, postgraduates – 77.8%, and faculty members – 78.5%), whereas the study of Ahmed *et al.* revealed that only half of the law students (50%) acknowledged the same in regards to the protection of IPR [19]. This indicates that protection and laws of IPR are not known explicitly by the dental task force as it is not taught in the dental education process. Furthermore, interns (55.3%) and postgraduates (48.3%) were less informed than faculty members (68.4%) and other dental surgeons (100%) about the laws protecting the consumers. This might be possible as the staff and dental surgeons are well educated and informed about consumer protection as they might have attended seminars in the past. The alarming fact was that about 23.1% of interns, 33.9% of postgraduates and 32.3% of faculty members had been a victim of unfair trade practices. This might be due to the fact that dental professionals are not educated regarding fair trade practices. In regard to the base of the use of IPR, the majority (interns – 79.4%, postgraduates – 71.7%, faculty members – 73.1%, and other dental surgeons – 66.7%) thought that it might be sold, purchased, or registered. In comparison, only a few law students (27%) from the study conducted by Ahmed *et al.* agreed to this statement [19].

This study showed that very few interns (14.2%), postgraduates (7.2%), and faculty members (9.1%) had attended seminars/workshops related to IPR, and about 28.0% interns, 28.9% postgraduates, 36.4% faculty members, and 66.7% other dental surgeons wish to seek an impending need for IPR sensitization at their institute or faculty. The desire for sensitization might be due to a lack of IPR awareness among the dental task force. However, a great percentage of interns (87.3%), postgraduates (83.9%), faculty members (86.5%), and other dental surgeons (86.2%) wish to attend IPR seminars in the future. This could be a possibility as the dental health care professionals are not knowledgeable in regards to IPR due to the exclusion of IPR from their syllabus but have expressed great enthusiasm for participating in seminars relating to IPR. Therefore, the onset of imparting knowledge and education in the institution should be done at the earliest level of education by conducting lectures as it will prove to be beneficial for the interns and dental students who comprise the forthcoming dentists. The inclusion of IPR in student’s dental curriculum will highly increase and broaden their horizon. It is a widely accepted fact that in today’s industry, IPR has a major role to play.

The increasing importance given to oral health is due to the fact that oral health forms an integral, inseparable part of our general health. The fast-paced, ever-evolving dental education, dental procedures and treatment demand innovations from the dental fraternity. The ever-evolving sphere of research is gaining more popularity in all the institutions, not only among the postgraduates but also undergraduates and practicing dentists. Increasingly new techniques, formulations, procedures are being developed. However, due to the lack of awareness about IPR, these hard-earned findings are at risk of being poached and infringed. The knowledge about IPR is essential for the protection of innovation and research, paving the way beyond just the publications of these researches into patented armamentarium and treatment procedures.

The main limitation of our study was that it was conducted among the students of only private dental colleges of Navi Mumbai, excluding dental professionals. Such studies should be limited to the dental domain and have the future scope of being conducted across various health-related professionals. Studies may also be conducted to compare the awareness among these fraternities. Future studies might incorporate mixed-method analysis, a qualitative, quantitative hybrid approach, and a structured questionnaire conducted in a phased manner with focused group discussions, yielding targeted results. Training modules, continuing dental education programs, online training modules, conferences, and others may prove to be a valuable resource to increase sensitization about the various constructs of IPR.

## Conclusion

This study holds utmost importance in today’s dental scenario since it is a novel study about IPR. The information-based industry, including dental patent rights is gradually developing. IPR is an hour requirement and should be a part of competitive domestic and international trade. Without the distribution of IPR information and use, creating a creative environment is impossible. It is crucial for dental colleges to incorporate IPR into their basic education system, promoting IPR enrolment so that creators are protected, thereby encouraging new ideas. IPR should be reused in the content curriculum of dental colleges in order to improve its awareness. India has all the resources in terms of raw materials, cheap labor, creativity and dedication. There is no doubt that India and other developing countries will play an equal role in global trade through exploration and production in relation to intellectual property rights.

## Acknowledgements

### Ethical approval

The approval for this study was obtained from the Institutional Ethical Committee (Protocol Approval Number: TOC EC/01/2020).

### Consent to participate

Necessary permissions were obtained from the institutional and department heads of the participating colleges and the study was scheduled as per their convenience.

### Conflict of interest

The authors declare that there is no conflict of interest.
